# Neoadjuvant chemotherapy for breast cancer: Pathologic response rates but not tumor size, has an independent prognostic impact on survival

**DOI:** 10.1002/cam4.6930

**Published:** 2024-02-07

**Authors:** Gilles Houvenaeghel, Alexandre de Nonneville, Monique Cohen, Laura Sabiani, Max Buttarelli, Emmanuelle Charaffe, Aurélie Jalaguier, Marie Bannier, Agnès Tallet, Frédéric Viret, Anthony Gonçalves

**Affiliations:** ^1^ Department of Surgical Oncology Aix‐Marseille University, CNRS, INSERM, Institute Paoli‐Calmettes, CRCM Marseille France; ^2^ Department of Medical Oncology Aix‐Marseille University, CNRS, INSERM, Institute Paoli‐Calmettes, CRCM Marseille France; ^3^ Department of Pathology Aix‐Marseille University, CNRS, INSERM, Institute Paoli‐Calmettes, CRCM Marseille France; ^4^ Department of Radiology Aix‐Marseille University, CNRS, INSERM, Institute Paoli‐Calmettes, CRCM Marseille France; ^5^ Department of Radiotherapy Aix‐Marseille University, CNRS, INSERM, Institute Paoli‐Calmettes, CRCM Marseille France

**Keywords:** breast cancer, neoadjuvant chemotherapy, pathologic response, survival

## Abstract

**Aim:**

We investigated the pathologic complete response rates (pCR) and survival outcomes of early breast cancer patients who underwent neoadjuvant chemotherapy (NAC) over 14 years at a French comprehensive cancer center and reported pCR and survival outcomes by tumor subtypes and size.

**Methods:**

From January 2005 to December 2018, 1150 patients receiving NAC were identified. Correlations between cT stage, breast tumor response, axillary lymph node response, pCR, surgery, and outcomes were assessed. pCR was defined as (ypT0/ypTis) and (ypN0/pN0sn).

**Results:**

A pCR was reached in 31.7% (365/1150) of patients and was strongly associated with tumor subtypes, but not with tumor size (pretreatment cT category). Luminal‐B Her2‐negative and triple‐negative (TN) subtypes, cN1 status, older age, and no‐pCR had an independent negative prognostic value. Overall survival (OS), relapse‐free survival (RFS), and metastasis‐free survival (MFS) were not significantly different for cT0‐1 compared to cT2 stages. In Cox‐model adjusted on in‐breast pCR and pN status, ypN1 had a strong negative impact (OS, RFS, and MFS: HR = 3.153, 4.677, and 6.133, respectively), higher than no in‐breast pCR (HR = 2.369, 2.252, and 2.323). A negative impact of no pCR on OS was observed for cN0 patients and TN tumors (HR = 4.972) or HER2‐positive tumors (HR = 11.706), as well as in Luminal‐B Her2‐negative tumors on MFS (HR = 2.223) and for Luminal‐A on RFS (HR = 4.465) and MFS (HR = 4.185).

**Conclusion:**

Achievement of pCR, but not tumor size (pretreatment cT category), has an independent prognostic impact on survival. These results suggest potential NAC benefits in patients with small tumors (<2 cm), even in absence of clinically suspicious lymph nodes. Residual lymph node disease after NAC is the most powerful adverse prognostic factor.

## 1 INTRODUCTION

According to the most recent data of global cancer statistics 2020, breast cancer (BC) has an estimated incidence of 2.3 million new cases and has become the most diagnosed cancer, while being the fifth most lethal tumor type with 685,000 deaths.^1^ Fortunately, mortality has been constantly decreasing during the last 20 years, mostly due to early diagnosis and progress in therapeutics, including systemic treatment such as cytotoxic chemotherapy.

During the past decades, it has been demonstrated by several randomized clinical trials, as well as meta‐analyses, that chemotherapy in operable BC provides a similar survival benefit when administered either after or before surgical resection.^2^, ^3^, ^4^, ^5^, ^6^ In addition, neoadjuvant chemotherapy (NAC) increases the rate of breast‐conserving surgery. Consequently, NAC has become increasingly popular in operable BC, when initial breast conservation is deemed to be not feasible, but also in smaller tumors, notably in subtypes in which adjuvant chemotherapy is almost always necessary and the probability of response is the highest, such as HER2‐positive and triple‐negative BC (TNBC). Moreover, accumulating pieces of evidence demonstrated that the achievement of pathological complete response (pCR) after NAC represents a strong favorable prognostic factor for survival,^7^, ^8^, ^9^ which is particularly relevant in the latter subtypes. Importantly, recent results from randomized trials have shown that patients not reaching pCR may benefit from adjuvant postoperative systemic treatments, including trastuzumab emtansine in HER2‐positive BC,^10^ capecitabine in TNBC,^11^ or olaparib^12^ in germline BRCA‐mutated BC. Recently, pembrolizumab addition to neoadjuvant and adjuvant therapy has become a standard for T2 and/or N‐positive TNBC, based on the Keynote 522 study results.^13^, ^14^ Accordingly, the use of NAC in these subtypes should rapidly increase and become dominant, even in patients with relatively low tumor burden.

Most previous studies examining the relationships between pCR and outcomes were performed in patient populations receiving NAC in the context of large tumors in which initial breast‐conserving surgery was not recommended. Thus, it is unclear whether similar benefits may be found in smaller tumors. In a previous study,^15^ we have shown that the pCR rate after NAC was neither higher nor significantly different for tumors <2 cm compared with tumors ≥2 cm, especially for HER2+ and TN subtypes for which adjuvant therapy can be offered to improve prognosis. An evaluation of OS and RFS comparing patients with these sizes of tumors and adjusted for other prognostic factors was warranted. In the present study, we aimed to examine correlations between pCR and survival outcomes according to tumor subtypes and tumor size (pretreatment cT category).

## 2 METHODS

### 2.1 Patient selection and study design

We retrospectively analyzed the medical records of early BC patients treated at the Institut Paoli‐Calmettes Comprehensive Cancer Center between January 2005 and December 2018. The patient data's were identified from clinical databases. This study was approved by our Clinical Research Department under the reference NAC‐TS‐IPC 2021–026; (ClinicalTrials.gov‐NCT02869607). Patient and tumor characteristics, pathological results, periods, and treatments, were collected. The present study included 1150 patients, who received NAC as their first treatment for non‐metastatic disease. NAC included a sequential combination of anthracyclines and taxanes. Patients with pN1sn without axillary lymph node dissection (ALND) after NAC (*n* = 28) and patients without axillary surgery (*n* = 8) were excluded (Figure S1). All patients with HER2‐positive disease received a trastuzumab‐based regimen during NAC and as adjuvant treatment. No patients received postoperative trastuzumab emtansine or capecitabine, which was either not available in this indication or not recommended at our institution at the time of treatment for the considered cohort of patients.

Patients receiving NAC were staged using clinical examination, ultrasonography, mammography, and breast MRI. Search for distant metastases using either a PET‐scan or a combination of CT scan and bone scan. Pretreatment sentinel lymph node biopsy (SLNB) with or without completion of ALND at the time of surgery or only ALND was used to determine lymph node status. SLNB was performed before NAC in patients with cN0 and cT ≤5 cm. Detection of SN was based on either combined technique or isotopic‐only detection during the last years. Three periods were determined: Period 1 (P1) 2005–2009, period 2 (P2) 2010–2014, and period 3 (P3) 2015–2018.

The determination of endocrine receptors (ER) and HER2 status was done according to French guidelines (ER positivity was determined by immunohistochemistry [IHC] with a 10% threshold for estrogen receptor and/or progesterone receptor positivity. HER2 status was determined based on an IHC HER2 score of 3+ and/or HER2 amplification by in situ hybridization). Tumor subtypes were defined based on tumor grade, ER status, and HER2 status, resulting in five surrogate molecular subtypes: Luminal A‐like (ER+/HER2−/Grade 1 or 2), Luminal B‐like HER2‐negative (ER+/HER2−/Grade 3), Luminal B‐like HER2‐positive (ER+/HER2+), HER2‐positive (ER−/HER2+), and TN (ER−/HER2‐).^16^ We examined the association of various criteria with different time periods. Specifically, we assessed the association of cT0–1 stage (cT0 tumors being clinically non‐palpable lesions that may correspond to ultrasound sizes of usT1 or usT2) or cT2‐3‐4, ypT0‐is or ypT ≥1, pCR or no pCR, and breast conservative surgery or mastectomy. We conducted both univariate and multivariate analyses using binary logistic regression to determine significant criteria identified in the univariate analysis. For the assessment of breast pCR, patients with residual components of ductal carcinoma in situ were considered to have achieved pCR according to the National Surgical Adjuvant Breast and Bowel Project criteria.^17^ Patients with residual lymph node tumor in the axillary region (ypN1) were considered not to have achieved pCR. pCR was defined as ypT0 or ypTis with ypN0 or pN0sn. Overall survival (OS), recurrence‐free survival (RFS), and metastasis‐free survival (MFS) were defined as the time interval from the date of diagnosis to death or last follow‐up, to recurrence or last follow‐up, and distant recurrence or last follow‐up, respectively. Patients lost to follow‐up were considered as alive as of the date of the last contact.

### 2.2 Statistics

The associations between categorical values were evaluated via chi‐squared tests. We calculated survival functions using the Kaplan–Meier method and assessed differences using the log‐rank test. For the multivariate survival analyses, we employed the Cox proportional‐hazard regression model and adjusted for significant variables identified in the univariate analysis. We determined hazard ratio (HR), confident interval 95% (CI 95%), and *p*‐value. Statistical significance was set as *p* ≤ 0.05. Analyses were performed with SPSS‐16.0 (SPSS‐Inc., Chicago‐Illinois, USA).

## 3 RESULTS

### 3.1 Patient characteristics

The median age of all patients (*n* = 1150) was 50 years old (CI 95% 49.7–51.1). Characteristics of patients according to cT stage are reported in Table S1. Most of the patients had cT2 (57.4%) or cT3 (24.9%) stage and more than half had cN1 disease (55.7%). Forty patients had non clinically palpable tumors (cT0), including 16 cT0 cN0, 21 cT0 cN1, and 3 cT0 cN2 cases. Complete mastectomy was performed in 57.3% of cases and most patients had ALND (86.2%). The vast majority of patients with negative pretreatment SNL biopsy had no ALND (156 out of 161). Almost 60% of patients had HER2‐positive (29.5%) or TN (28.9%) disease. The distribution of cT stages was significantly different according to cN stage, breast surgery, axillary surgery, pathological axillary nodal status, and periods. Axillary surgeries for all patients and cN0 patients only are reported in Table S2. Among patients with lymph node involvement, 62.6% had one to three positive lymph nodes, and 37.4% had more than three. All factors analyzed (periods, tumor subtypes, cN stage, cT stage, and breast surgery) were significantly associated with axillary surgery. Thus, the use of SLNB‐only increased with time (from 1.1% during P1 to 21.2% during P3), while ALND with or without previous SLNB was more common in larger or cN+ tumors. It was also predominant in Luminal subtypes, while exclusive SLNB was more frequent in TN subtypes. Similar results were found in patients with cN0 stage.

### 3.2 Pathologic response

Among all patients, pCR rate was 31.7% (365/1150) with significantly different rates according to tumor subtypes: 7.1%, 9.6%, 21.9%, 23.0%, and 38.1% for Luminal A, Luminal B‐like HER2‐negative, Luminal B‐like HER2‐positive, HER2‐positive, and TN tumors, respectively, and with significant rates according to cN stages (35.9% and 28.7% for cN0 and cN1, respectively) (Table 1). Other factors (cT stages and periods) had no significant impact on pCR. For Luminal A tumors, pCR rates were 3.3% and 9.3% for Grade 1 and Grade 2 tumors, respectively, but the difference was not significant (*p* = 0.189).

**TABLE 1:** pCR rates according to tumor subtypes, cT stages, cN stages, periods and grade for Luminal A tumors.

	pCR		No pCR		Chi‐squared test
	Nb	%	Nb	%	*p*‐value
All patients	365	31.7	785	68.3	
Period					
P1	93	25.5	183	23.3	0.486
P2	147	40.3	345	43.9	
P3	125	34.2	257	32.7	
T subtypes					
Luminal A	26	7.1	293	37.3	<0.0001
Luminal B‐like HER2‐negative	35	9.6	122	15.5	
Luminal B‐like HER2‐positive	80	21.9	113	14.4	
HER2‐positive	84	23.0	62	7.9	
Triple Negative	139	38.1	193	24.6	
Luminal HER2‐negative Grade?	1	0.3	2	0.3	
cT stage					
cT0‐1	37	10.1	95	12.1	0.141
cT2	226	61.9	434	55.3	
cT3	78	21.4	208	26.5	
cT4	24	6.6	48	6.1	
cN stage					
cN0	175	47.9	313	39.9	0.036
cN1	184	50.4	457	58.2	
cNx	6	1.6	15	1.9	
Luminal A Grade 1	2	7.7	58	19.8	0.189
Luminal A Grade 2	24	92.3	235	80.2	

Abbreviations: cT, clinical tumor size stage; cN, clinical lymph node status; pCR, pathologic complete response.

Examining pCR rates in each subtype for both cN0 and cN1 stages revealed that there was no significant difference in pCR according to cT stage. (Table S3).

Among 377 patients with in‐breast pCR and axillary surgery after NAC, 23.1% (87/377) had residual nodal burden: 50.0%, 31.1%, 16.9%, 12.3%, and 20.8% for Luminal A, Luminal B‐like HER2‐negative, Luminal B‐like HER2‐positive, HER2‐positive, and TN tumors, respectively (*p* < 0.0001) (Table S4). Among 612 patients with no in‐breast pCR and axillary surgery after NAC, 37.1% (227/612) had no residual nodal burden: 28.5%, 35.35%, 49.4%, 40.4%, and 45.8% for Luminal A, Luminal B‐like HER2‐negative, Luminal B‐like HER2‐positive, Her2‐positive, and TN tumors, respectively (Table S4).

### 3.3 Survival outcomes in the overall population

Median follow‐up was 66.27 months (mean: 70.08, CI 95% 67.88–72.28, range 4.38–197.5). OS, RFS, and MFS rates with the number of patients at risk and the number of events are reported in Table S5. During the follow‐up, there were 196 deaths (179 from BC evolution, 13 from other cancers, and 4 from other causes), 263 metastasis, 44 local recurrences (3.5%: 17/491 after BCS and 4.1%: 27/659 after mastectomy), and 37 nodal recurrences. The site of the first metastatic relapse was: 72 multiple localizations, 52 bone metastasis, 37 liver metastasis, 33 cerebral metastasis, 24 pulmonary metastasis, 20 distant nodal metastasis, 13 skin metastasis, 9 peritoneal metastasis, and 3 others (localization unknown).

By univariate analysis, all factors tested, including age, cT, and cN stages, pathologic nodal status, and pCR, except periods of treatment, were significantly associated with OS, RFS, and MFS (Table S6).

**FIGURE 1:**
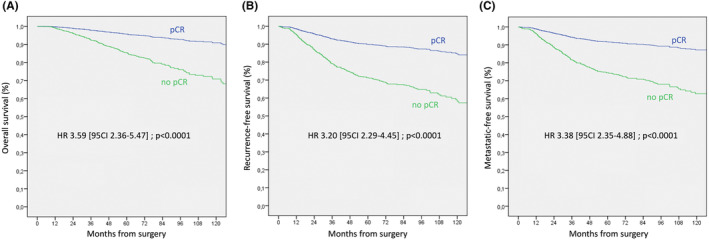
(A) Overall survival (OS), (B) recurrence free survival (RFS), and (C) metastases free survival (MFS) according to pCR in multivariate analysis. HR, Hazard ratio; pCR, pathologic complete response.

By multivariate analysis (Table 2), OS, RFS, and MFS were not significantly different for Stage cT0‐1 in comparison with cT2 stage. A negative prognostic impact was significant for stages cT3 and cT4 in comparison with cT0‐1 stage, for OS (HR: 2.069 and 2.434, respectively), RFS (HR: 1.677 and 2.105), and MFS (HR: 1.884 and 2.041). No difference was also observed when size was analyzed as a continuous variable (data available for 1009 patients). Luminal B‐like HER2‐negative tumors, TN tumors, cN1 status, age, and no pCR (Figure 1) retained a significant independent adverse prognostic impact on OS, RFS, and MFS.

**TABLE 2:** Survival results in multivariate analysis.

Cox regression analysis		OS			RFS			MFS		
		HR	CI 95%	*p‐value*	HR	CI 95%	*p*	HR	CI 95%	*p‐value*
Adjusted on pCR										
Tumor size										
cT0‐1		1			1			1		
cT2		1.255	0.771–2.381	0.291	1.396	0.903–2.160	0.133	1.541	0.953–2.452	0.081
cT3		2.069	1.153–3.714	0.015	1.677	1.055–2.664	0.029	1.884	1.133–3.135	0.015
cT4		2.434	1.179–5.025	0.016	2.105	1.178–3.763	0.012	2.041	1.075–3.878	0.029
cN status										
cN0		1			1			1		
cN ≥1		1.691	1.224–2.335	0.001	1.609	1.243–2.082	<0.0001	1.718	1.303–2.265	<0.0001
cNx		1.419	0.547–3.684	0.472	1.521	0.686–3.372	0.302	1.788	0.802–3.987	0.156
pCR										
pCR		1			1			1		
No pCR		3.589	2.356–5.466	<0.0001	3.196	2.293–4.454	<0.0001	3.381	2.345–4.875	<0.0001
Subtypes										
Luminal A		1			1			1		
Luminal B‐like HER2‐negative		1.865	1.213–2.867	0.005	1.460	1.028–2.075	0.035	1.449	1.012–2.075	0.043
Luminal B‐like HER2‐positive		0.751	0.416–1.355	0.341	0.921	0.611–1.388	0.693	0.797	0.511–1.244	0.318
HER2‐positive		1.564	0.893–2.742	0.118	1.576	1.035–2.400	0.034	1.403	0.895–2.198	0.139
Triple negative		3.372	2.310–4.823	<0.0001	1.965	1.444–2.676	<0.0001	1.729	1.251–2.390	0.001
Age										
≤40		1			1			1		
41–49		1.779	1.112–2.845	0.016	1.246	0.872–1.779	0.227	1.318	0.903–1.922	0.152
50–74		2.028	1.320–3.117	0.001	1.473	1.068–2.031	0.018	1.475	1.046–2.082	0.027
>74		8.487	4.202–17.14	<0.0001	2.525	1.265–5.044	0.009	2.356	1.094–5.074	0.029
Adjusted on in‐breast pCR and pN										
Tumor size										
cT0‐1		1			1			1		
cT2		1.275	0.720–2.257	0.404	1.344	0.863–2.091	0.190	1.473	0.902–2.405	0.121
cT3		1.956	1.080–3.543	0.027	1.572	0.983–2.514	0.059	1.747	1.044–2.924	0.034
cT4		2.134	1.029–4.423	0.042	1.851	1.032–3.321	0.039	1.791	0.939–3.415	0.077
cN status										
cN0		1			1			1		
cN ≥1		1.360	0.956–1.935	0.087	1.203	0.909–1.592	0.196	1.239	0.919–1.671	0.160
cNx		1.151	0.444–2.982	0.772	1.219	0.551–2.699	0.625	1.377	0.619–3.066	0.433
Subtypes										
Luminal A		1			1			1		
Luminal B‐like HER2‐negative		2.028	1.323–3.110	0.001	1.547	1.089–2.199	0.015	1.537	1.074–2.202	0.019
Luminal B‐like HER2‐positive		0.880	0.485–1.596	0.674	1.087	0.718–1.647	0.693	0.945	0.603–1.480	0.804
HER2‐positive		1.743	0.971–3.007	0.054	1.866	1.219–2.857	0.004	1.674	1.062–2.638	0.026
Triple negative		4.221	2.847–6.258	<0.0001	2.518	1.831–3.462	<0.0001	2.259	1.618–3.153	<0.0001
Age										
≤40		1			1			1		
41–49		1.734	1.084–2.773	0.022	1.209	0.847–1.728	0.296	1.274	0.873–1.858	0.210
50–74		1.999	1.300–3.073	0.002	1.410	1.022–1.947	0.036	1.404	0.994–1.983	0.054
>74		7.951	3.928–16.09	<0.0001	2.298	1.149–4.597	0.019	2.133	0.988–4.605	0.054
pN										
pN0sn		1			1			1		
ypN0		1.635	0.818–3.271	0.164	2.434	1.313–4.512	0.005	3.105	1.479–6.520	0.003
ypN1		3.153	1.543–6.444	0.002	4.677	2.479–8.821	<0.0001	6.133	2.876–13.08	<0.0001
In‐breast	Yes	1			1			1		
pCR	No	2.369	1.623–3.458	<0.0001	2.252	1.662–3.051	<0.0001	2.323	1.672–3.227	<0.0001

Abbreviations: cN, clinical lymph node status; cT, clinical tumor size stage; MFS, metastases free survival; OS, Overall survival; pCR, pathologic complete response; RFS, recurrence free survival.

In a Cox model adjusted on in‐breast pCR and pN status, ypN1 status had a strong negative impact on survival (OS, RFS, and MFS: HR: 3.153, 4.677, and 6.133, respectively), higher than no in‐breast pCR (HR: 2.369, 2.252, and 2.323 for OS, RFS, and MFS, respectively). A negative prognosis impact was observed on MFS for ypN0 status (HR: 3.105 for MFS and 2.434 for RFS) in comparison with pN0sn status: 52.77% of ypN0 patients were cN1 stage. To note that when introducing periods in the co‐variables to assess if the variation in management of the axilla over time might be confounding, consistent results were observed.

### 3.4 Survival outcomes in sub‐groups of interest (Table 3)

**TABLE 3:** Survival results in sub‐groups of interest.

	OS			RFS			MFS		
	HR	CI 95%	*p‐value*	HR	CI 95%	*p*	HR	CI 95%	*p‐value*
Triple negative tumors cN0 (*n* = 156)									
cT stage									
cT0‐1	1			1			1		
cT2	0.295	0.087–1.001	0.050	0.575	0.185–1.786	0.338	0.837	0.181–3.870	0.820
cT3	0.911	0.245–3.392	0.889	1.260	0.359–4.416	0.718	2.169	0.427–11.02	0.351
cT4	2.056	0.216–19.57	0.531	2.025	0.214–19.11	0.538	3.360	0.288–39.13	0.333
Age									
≤40	1			1			1		
41–49	1.804	0.302–10.77	0.517	1.138	0.241–5.382	0.870	0.628	0.149–4.217	0.785
50–74	5.298	1.160–24.21	0.031	4.228	1.208–14.80	0.024	2.949	0.813–10.70	0.100
>74 (1 patient)									
pCR									
pCR	1			1			1		
No pCR	4.972	1.446–17.10	0.011	4.717	1.609–13.83	0.005	4.673	1.353–16.14	0.015
HER2‐positive tumors (ER+ or ER‐) cN0 (*n* = 136)									
cT stage									
cT0‐1	1			1			1		
cT2			0.960			0.939			0.951
cT3			0.955			0.942			0.951
cT4	0.645		0.999			0.999			0.999
age									
≤40	1			1			1		
41–49	0.184	0.014–2.447	0.200	0.660	0.158–2.746	0.567	2.470	0.264–23.09	0.428
50–74	0.327	0.039–2.735	0.302	0.395	0.096–1.614	0.196	1.065	0.108–10.51	0.957
>74 (3 patient)			0.991	3.661	0.351–37.92	0.277	8.972	0.506–159.1	0.135
pCR									
pCR	1			1			1		
No pCR	11.706	1.270–107.9	0.030	2.522	0.774–8.214	0.125	4.361	0.870–21.86	0.073
Patients ypT0 and ypTis (*n* = 452)^a^									
ypT0	1			1			1		
ypTis	1.076	0.480–2.410	0.859	1.187	0.657–2.146	0.570	1.231	0.649–2.337	0.525
Luminal B‐like HER2‐negative (cN0 & cN1) (*n* = 157)									
cT stage									
cT0‐1	1			1			1		
cT2	6.441	0.865–47.95	0.069	4.764	1.136–19.98	0.033	4.318	1.027–18.16	0.046
cT3	5.228	0.624–43.80	0.127	3.863	0.837–17.83	0.083	3.812	0.824–17.64	0.087
cT4	13.392	1.151–112.7	0.037	4.772	0.751–30.31	0.098	4.180	0.666–26.25	0.127
Age									
≤40	1			1			1		
41–49	0.973	0.380–2.492	0.955	0.668	0.297–1.503	0.330	0.633	0.276–1.452	0.281
50–74	1.082	0.453–2.584	0.859	1.031	0.518–2.054	0.930	0.967	0.483–1.938	0.925
>74	8.512	2.047–35.40	0.003	1.288	0.161–10.28	0.811	1.325	0.165–10.64	0.791
pCR									
pCR	1			1			1		
No pCR	1.688	0.684–4.168	0.256	2.057	0.908–4.659	0.084	2.223	0.931–5.310	0.072
cN status									
cN0	1			1			1		
cN ≥1	1.023	0.502–2.086	0.950	1.226	0.663–2.267	0.515	1.108	0.594–2.065	0.748
Luminal A (cN0 and cN1) (*n* = 319)									
cT stage									
cT0‐1	1			1			1		
cT2	0.698	0.255–1.916	0.486	0.550	0.273–1.108	0.094	0.578	0.278–1.201	0.142
cT3	1.479	0.542–4.032	0.445	1.036	0.506–2.121	0.923	1.143	0.544–2.399	0.724
cT4	1.000	0.234–4.280	1.000	1.276	0.501–3.250	0.609	1.252	0.466–3.366	0.656
Age									
≤40	1			1			1		
41–49	1.438	0.504–4.100	0.497	0.899	0.436–1.687	0.763	0.952	0.469–1.931	0.892
50–74	1.931	0.728–5.124	0.186	1.106	0.596–2.053	0.789	1.111	0.585–2.110	0.747
>74 (6 patients)	3.830	0.434–33.79	0.227	0.000	0.000‐	0.961	0.000	0.000‐	0.962
pCR									
pCR	1			1			1		
No pCR	2.229	0.532–9.331	0.273	4.465	1.083–18.41	0.038	4.185	1.014–17.27	0.048
cN status									
cN0	1			1			1		
cN ≥1	1.811	0.945–3.470	0.074	1.111	0.694–1.780	0.661	1.211	0.744–1.973	0.441
Grade									
1	1			1			1		
2	2.341	0.969–5.654	0.059	1.434	0.804–2.558	0.222	1.514	0.833–2.754	0.174

Abbreviations: cN, clinical lymph node status; cT, clinical tumor size stage; MFS, metastases free survival; OS, Overall survival; pCR, pathologic complete response; RFS, recurrence free survival; ypT, pathologic tumor status after neoadjuvant chemotherapy.

^a^ Adjusted on cT stage, age, cN stage, and tumor subtypes.

In patients with in‐breast pCR (*n* = 452), there was no significant difference in OS, RFS, and MFS between ypT0 and ypTis status after NAC in Cox regression analysis adjusted on cT stage, age, cN stage, and tumor subtypes.

**FIGURE 2:**
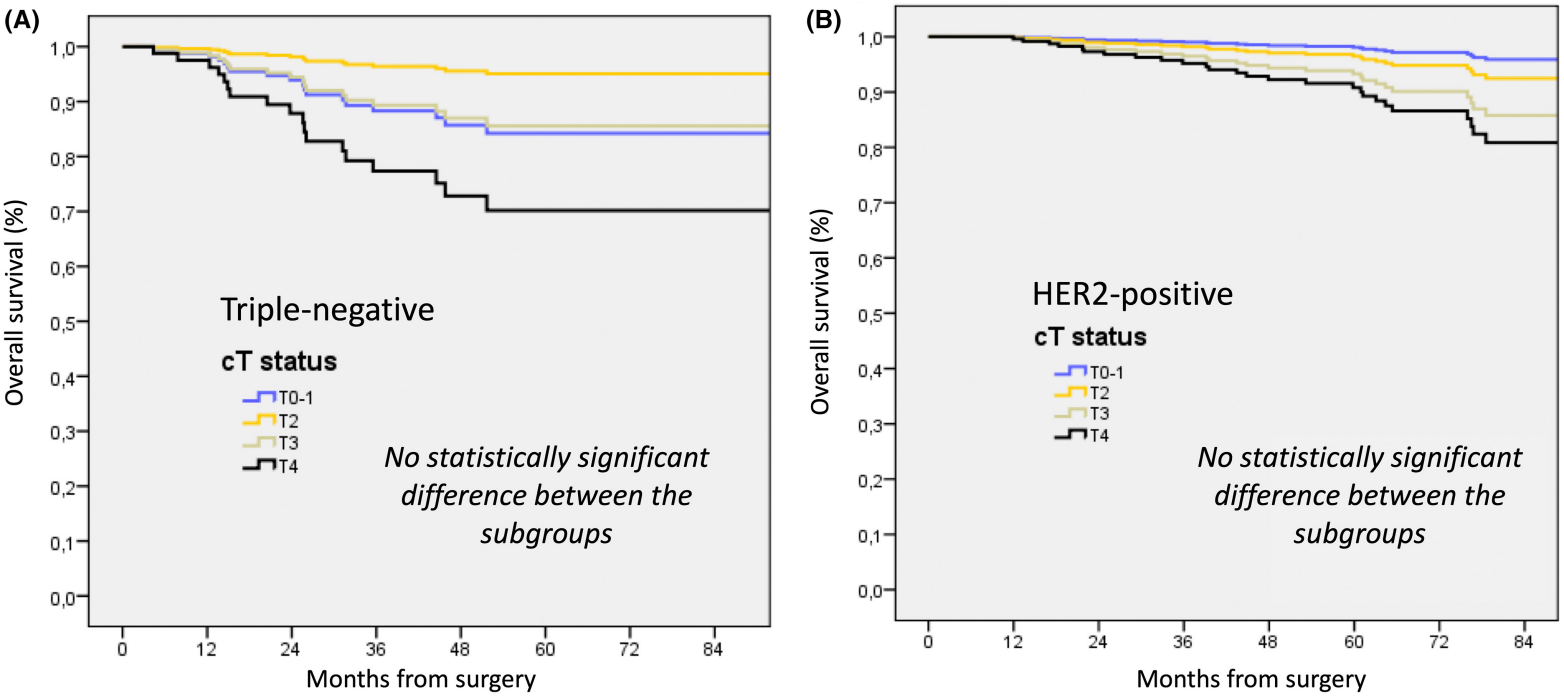
Overall survival according to cT stage in multivariate analysis for patients with cN0 triple negative (A) and Her2‐positive tumors (B).

In cN0 TN tumors (*n* = 156), a significant negative prognostic impact was observed for patients without pCR on OS, RFS, and MFS (HR: 4.972, 4.717, and 4.673, respectively). Age >50 years old was also associated with a significant negative prognosis impact for OS and RFS (HR: 5.298 and 4.228, respectively). No significant impact of cT stage (cT2, cT3, cT4) was observed in comparison to cT0–1 stage, except for patients with cT2 stage who had a better OS outcome (HR: 0.295) (Figure 2A).

In cN0 HER2‐positive tumors, either ER‐positive or ER‐negative (*n* = 136), a significant negative prognostic impact was observed for patients without pCR on OS (HR: 11.706). No other factors were significantly associated with survival outcome, particularly cT stage (cT2, cT3, and cT4) in comparison with cT0–1 stage (Figure 2B).

In Luminal B‐like HER2‐negative tumors, cN0 and cN1 (*n* = 157), a significant negative prognostic impact was observed for patients with cT2 tumors on RFS (HR: 4.764) and MFS (HR: 4.318) in comparison with cT0–1 tumors, and for cT4 tumors on OS (HR: 13.392). Other factors significantly associated with survival outcomes were age >74 years old for OS (HR: 8.512) and no pCR for MFS (HR: 2.223) and RFS (HR: 2.057), with borderline significance (*p* = 0.072 and *p* = 0.084, respectively).

In Luminal A‐like, cN0, and cN1 tumors (*n* = 319), a significant negative prognostic impact was observed for patients without pCR on RFS (HR: 4.465) and MFS (HR: 4.185) and for patients with cN1 status on OS (HR: 1.811) with borderline significance (*p* = 0.074). Of note, pretreatment cT category did not have any impact on any survival outcome. A negative prognostic impact with borderline significance on OS was suggested for Grade 2 tumors in comparison with Grade 1 tumors (HR: 2.341, *p* = 0.059).

## 4 DISCUSSION

In this monocentric retrospective study, we found that pCR rates were strongly associated with tumor subtypes, without significant differences according to cT stages, particularly between cT0‐1 and cT2. For patients with in‐breast pCR and axillary surgery after NAC, there was a significant association between ypN1 and tumor subtypes. An independent negative prognostic impact was observed for cT3 and cT4 stages in comparison with cT0‐1 stage, for OS, RFS, and MFS, but without a significant difference between cT0‐1 and cT2 stages. In a Cox model adjusted on in‐breast pCR and pN status, ypN1 status had a strong negative impact on OS, RFS, and MFS, which was greater than no in‐breast pCR. Looking separately at tumor subtypes, no negative prognostic impact on survival was reported for cT2 in comparison with cT0‐1 stage, except for Luminal B Her2‐negative subtypes. In contrast, the absence of pCR was associated with an adverse survival outcome (either OS, RFS or MFS, or both), and it was observed in all tumor subtypes.

Our results are in line with previous evidence showing a strong association between pCR rates and tumor subtypes: pCR was higher in HER2‐positive and TN subtypes, and lower in Luminal subtypes. However, a more counterintuitive result of the present study was the lack of a significant association between clinical tumor size (pretreatment cT category) and pCR rates. Data from existing literature on this point are relatively controversial. Von Minckwitz et al. reported the German experience on 6377 patients included in seven randomized trials evaluating NAC and found pCR rates (i.e., ypT0/is N0) of 25, 21.9, 14.2, 13.5, and 15.6% in cT1, cT2, cT3, cT4a–c, and cTl4d.^16^ While the difference between cT1 and cT2 was not significant, the pCR rates for T3 and T4 were lower. Similarly, in the meta‐analysis from Cortazar et al. including 11,955 patients, the pCR rates were comparable between cT1 and cT2 (18.3% vs. 19.9%) but seemed to be lower for cT3, T4a–c, and T4d (13.0%, 14.5%, and 16.0% respectively).^7^ In addition, examining 2366 patients from the Netherlands Cancer Registry receiving NAC, Goorts^18^ found pCR rates of 31, 22, 18, and 17% in cT1, cT2, cT3, and cT4, respectively. They concluded that the clinical T stage was the most important predictor of pCR. In the US prospective Neoadjuvant Breast Symphony Trial study enrolling 608 evaluable patients, the probability of pCR was decreased when tumor size was more than 5 cm, particularly in the Basal and HER2 subtypes. Yet, there was no statistically significant impact of pretreatment cT category on pCR rates by multivariate analysis in any subtype.^19^ In addition, in a monocentric retrospective US study including 366 patients with stage II and III disease treated with NAC, the association of lower clinical stage and probability of pCR had only borderline significance.^20^ Finally, in two additional studies focusing on TN tumors, there was no statistically significant impact of clinical stage on the probability of achieving pCR.^21^, ^22^ Thus, the present results confirm other data indicating that the probability of pCR may be more correlated with tumor biology than tumor size, at least in small‐or moderate‐sized tumors.

Regarding survival outcomes, they were also tightly associated with tumor subtypes, being inferior in Luminal B HER2‐negative and TN tumors compared with Luminal A tumors. Of note, there was no significant impact of HER2‐positive subtypes on survival, all these patients were treated with neoadjuvant and adjuvant trastuzumab, even though none received trastuzumab emtansine. Importantly, the absence of pCR was an independent predictor of survival observed in all subtypes, including Luminal A, the most chemo‐resistant subtype. These results are in contrast with those from the previously cited German meta‐analysis, in which there was no difference at all according to pCR status after NAC for Luminal A subtypes. Again, in the meta‐analysis from Cortazar et al.,^7^ the difference was also non‐significant between pCR and no‐pCR status after NAC in hormone receptor‐positive/HER2‐negative tumors with Grades 1–2, even though the hazard ratio was numerically favorable to patients reaching pCR (HR 0.63, 95% CI 0,38–1,04). The reasons for such a discordance are unclear, the definition of subtypes being similar in the present study and these previous analyses. Another intriguing result was that, even though clinical stage (i.e., cT3 and cT4, or cN1) was independently associated with worse survival, there was no statistically significant difference in survival between cT0–1 and cT2 stages, notably in HER2‐positive and TN subtypes, but also in Luminal A subtype. The only exception was in Luminal B‐like HER2‐negative tumors, in which survival was significantly lower in cT2 versus cT0–1. It may suggest that in this latter subtype, and oppositely to endocrine‐sensitive Luminal A tumors, mechanisms of resistance to endocrine therapy may be favored in larger tumors ultimately leading to decrease survival, independently of chemo‐sensitivity.

For a long time, NAC has essentially aimed to decrease mastectomy rate for patients with locally advanced and large BCs.^23^ In the case of patients with HER2‐positive and TN tumors, the determination of whether they achieve pCR can help guide the selection of postoperative treatments, resulting in a significant reduction in the risk of recurrence. This has been reinforced by the generalization of trastuzumab emtansine for HER2‐positive tumors and capecitabine for TN tumors in the adjuvant setting in case of absence of pCR. As a result, recent guidelines suggest that NAC should be recommended for TN and HER2‐positive BC larger than 2 cm in size.^24^ Our results showing that pCR and survival rates correlated with intrinsic tumor features and clinical lymph node status rather than clinical pretreatment tumor size may support even broader use of NAC in these subtypes, including cT1 and sometimes cT1b with cN1 or pN1sn when SLNB is performed before NAC. Like others,^25^ we reported a strong negative survival impact of residual nodal tumor burden (ypN1), and completion of ALND is recommended for SLN micro and macro metastases after NAC.^26^ Moreover, for cN1 patients, the highest axillary pCR rate was reported for ER‐negative/Her2‐positive tumor subtype, but without major differences in axillary pCR rates per tumor subtype.^27^

Despite the inclusion of a substantial number of early BC patients who underwent NAC over a span of 14 years, our study has several limitations that need to be acknowledged. These limitations primarily stem from the retrospective nature of the study, including the possibility of selection bias and the absence of standardized treatment protocols. A centralized pathology review was not conducted for all cases. It is important to note that, while the definition of Luminal subtypes considered tumor grade, it did not incorporate the assessment of KI67. Additionally, no patients included in this study received adjuvant postoperative systemic treatments, such as trastuzumab emtansine in HER2‐positive BC, capecitabine in TNBC, or olaparib in germline BRCA‐mutated TNBC. No perioperative pembrolizumab was administrated at the time of the study for T2 and/or N+ patients.

## 5 CONCLUSION

There were no notable differences or significant variations in the pCR rate and survival outcomes after NAC between tumors smaller than 2 cm and tumors 2 cm or larger. This observation particularly applies to HER2‐positive and TN subtypes, for which adjuvant therapy can be provided to enhance prognosis. The pCR rate and survival outcomes seem to be associated more closely with intrinsic tumor characteristics and the clinical status of lymph nodes, rather than pretreatment cT category. These findings suggest that offering NAC to patients with these tumor subtypes, even in the absence of clinically suspicious lymph nodes (cN0) when the tumor is smaller than 2 cm, may potentially yield benefits.

## AUTHOR CONTRIBUTIONS

**Gilles Houvenaeghel:** Conceptualization (equal); data curation (equal); formal analysis (equal); methodology (equal); project administration (equal); resources (equal); supervision (equal); validation (equal); writing – original draft (equal); writing – review and editing (equal). **Alexandre de Nonneville:** Project administration (supporting); writing – original draft (equal); writing – review and editing (equal). **Monique Cohen:** Resources (equal); writing – review and editing (equal). **Laura Sabiani:** Resources (equal); writing – review and editing (equal). **Max Buttarelli:** Resources (equal); writing – review and editing (equal). **Emmanuelle Charaffe:** Resources (equal); writing – review and editing (equal). **Aurélie Jalaguier:** Resources (equal); writing – review and editing (equal). **Marie Bannier:** Resources (equal); writing – review and editing (equal). **Agnès Tallet:** Resources (equal); writing – review and editing (equal). **Frédéric Viret:** Resources (equal); writing – review and editing (equal). **Anthony Gonçalves:** Resources (equal); validation (equal); writing – original draft (equal); writing – review and editing (equal).

## FUNDING INFORMATION

This academic work did not receive financial support from any funding source.

## CONFLICT OF INTEREST STATEMENT

Alexandre de Nonneville declares Consulting fees by Gilead, Seagen, Lilly, and Novartis, payment or honoraria for lectures, presentations, speakers bureaus, manuscript writing, or educational events by Gilead, Daiichi Sankyo, and MSD, Support for attending meetings and/or travel by Gilead, Lilly, and Daiichi Sankyo. Emmanuelle Charaffe declares payment or honoraria for lectures, presentations, speakers bureaus, manuscript writing, or educational events by Exact Science (to institution), Veracyte (to institution), and AstraZeneca. Anthony Gonçalves declares Support for attending meetings and/or travel by Menarini, Participation on a Data Safety Monitoring Board or Advisory Board by Novartis, MSD, and Daiichi Sankyo. No conflict of interest was declared by others authors.

## ETHICS STATEMENT

All procedures performed in this study involving human participants were done following the French ethical standards and with the 2008 Helsinki declaration. This study was approved by our Clinical Research Department under the reference NAC‐TS‐IPC 2021–026; (ClinicalTrials.gov‐NCT02869607), Informed consent was waived since all data were de‐identified and collected retrospectively from each center.

## Supporting information


Table S1.
Table S2.
Table S3.
Table S4.
Table S5.
Table S6.
Figure S1.

## Data Availability

Data are available upon reasonable request. Requests to access these datasets should be directed to houvenaeghelg@ipc.unicancer.fr.
