# Supplementary material for: Neoadjuvant chemotherapy for breast cancer: Pathologic response rates but not tumor size, has an independent prognostic impact on survival
Source: Cancer Med. 2024 Feb 7;13(3):e6930. doi: 10.1002/cam4.6930 (PMC10904968; doi:10.1002/cam4.6930)
Supplement: Supplementary file 1 — Table S1. Table S2. Table S3. Table S4. Table S5. Table S6. Figure S1. [file CAM4-13-e6930-s001.docx]

Supplementary Table 1: Characteristics of patients according to cT stage. Abbreviations: cT, clinical tumor size stage; cN, clinical lymph node status; SLNB, sentinel lymph node biopsy; ALND, axillary lymph node dissection; ypT, pathologic tumor status after neoadjuvant chemotherapy; pN, pathologic lymph node status; pCR, pathologic complete response.

Supplementary Table 2: Axillary surgery for all patients and cN0 patients only. Abbreviations: cT, clinical tumor size stage; cN, clinical lymph node status; SLNB, sentinel lymph node biopsy; ALND, axillary lymph node dissection.

Supplementary Table 3: pCR rates according to tumor subtypes combined with cT and cN stages. Abbreviations: cT, clinical tumor size stage; cN, clinical lymph node status; pCR, pathologic complete response.

Supplementary Table 4: Pathologic breast tumor response (ypT0-is) according to pN status, for all patients and for each tumor subtypes. Abbreviations: ypT, pathologic tumor status after neoadjuvant chemotherapy; ypN, pathologic nodal status after neoadjuvant chemotherapy; pN, pathologic lymph node status.

Supplementary Table 5: OS, RFS and MFS rates with number of patients at risk and number of events. Abbreviations: OS, Overall survival; RFS, recurrence free survival; MFS, metastases free survival; SE, standard error; Nb, number.

Supplementary Table 6: Survival results in univariate analysis. Abbreviations: OS, Overall survival; RFS, recurrence free survival; MFS, metastases free survival; cT, clinical tumor size stage; cN, clinical lymph node status; pCR, pathologic complete response; ypT, pathologic tumor status after neoadjuvant chemotherapy; ypN, pathologic nodal status after neoadjuvant chemotherapy; pN, pathologic lymph node status.

Supplementary Table 1: Characteristics of patients according to cT stage. Abbreviations: cT, clinical tumor size stage; cN, clinical lymph node status; SLNB, sentinel lymph node biopsy; ALND, axillary lymph node dissection; ypT, pathologic tumor status after neoadjuvant chemotherapy; pN, pathologic lymph node status; pCR, pathologic complete response.

|  |  | **cT0-1** |  | **cT2** |  | **cT3** |  | **cT4** |  | **Chi2** | **Total** |  |
| --- | --- | --- | --- | --- | --- | --- | --- | --- | --- | --- | --- | --- |
|  |  | Nb | % | Nb | % | Nb | % | Nb | % | *p-value* | Nb | % |
| Age | median | 52.0 |  | 49.0 |  | 49.0 |  | 55.0 |  |  | 50.0 |  |
|  | CI 95% | 49.3-53.6 | | 49.3-51.2 | | 48.0-50.7 | | 51.8-58.2 | |  | 49.7-51.1 | |
| cN stage | cN0 | 67 | 50.8 | 317 | 48.0 | 93 | 32.5 | 11 | 15.3 | <0.0001 | 488 | 42.4 |
|  | cN1 | 65 | 49.2 | 333 | 50.5 | 184 | 64.3 | 59 | 81.9 |  | 641 | 55.7 |
|  | cNx | 0 | 0 | 10 | 1.5 | 9 | 3.1 | 2 | 2.8 |  | 21 | 1.8 |
| Breast | Conservative | 70 | 53.0 | 348 | 52.7 | 65 | 22.7 | 8 | 11.1 | <0.0001 | 491 | 42.7 |
| surgery | Mastectomy | 62 | 47.0 | 312 | 47.3 | 221 | 77.3 | 64 | 88.9 |  | 659 | 57.3 |
| Axillary | SLNB | 24 | 18.2 | 124 | 18.8 | 11 | 3.8 | 0 | 0 | <0.0001 | 159 | 13.8 |
| surgery | ALND | 87 | 65.9 | 368 | 55.8 | 239 | 83.6 | 71 | 98.6 |  | 765 | 66.5 |
|  | SLNB+ALND | 21 | 15.9 | 168 | 25.5 | 36 | 12.6 | 1 | 1.4 |  | 226 | 19.7 |
| Subtypes | Luminal A | 41 | 31.1 | 173 | 26.2 | 85 | 29.7 | 20 | 27.8 | 0.260 | 319 | 27.7 |
|  | Luminal B-like HER2-negative | 20 | 15.2 | 89 | 13.5 | 38 | 13.3 | 10 | 13.9 |  | 157 | 13.7 |
|  | Luminal B-like HER2-positive | 20 | 15.2 | 115 | 17.4 | 47 | 16.4 | 11 | 15.3 |  | 193 | 16.8 |
|  | HER2-positive | 12 | 9.1 | 84 | 12.7 | 35 | 12.2 | 15 | 20.8 |  | 146 | 12.7 |
|  | Triple Negative | 37 | 28.0 | 189 | 30.0 | 81 | 28.3 | 16 | 22.2 |  | 332 | 28.9 |
|  | Luminal HER2-negative Grade? | 2 | 1.5 | 1 | 0.2 | 0 | 0 | 0 | 0 |  | 3 | 0.3 |
| ypT | ypT0 | 44 | 33.3 | 202 | 30.6 | 86 | 30.1 | 24 | 33.3 | 0.334 | 356 | 31.0 |
|  | ypTis | 10 | 7.6 | 66 | 10.0 | 16 | 5.6 | 4 | 5.6 |  | 96 | 8.3 |
|  | ypT >= 1 | 78 | 59.1 | 392 | 59.4 | 184 | 64.3 | 44 | 61.1 |  | 698 | 60.7 |
| pN | pN0sn | 25 | 18.9 | 124 | 18.8 | 12 | 4.2 | 0 | 0 | <0.0001 | 161 | 14.0 |
|  | ypN0 | 48 | 36.4 | 298 | 45.2 | 137 | 47.9 | 34 | 47.2 |  | 517 | 45.0 |
|  | ypN1 | 59 | 44.7 | 238 | 36.1 | 137 | 47.9 | 38 | 52.8 |  | 472 | 41.0 |
| pCR | Yes | 37 | 28.0 | 226 | 34.2 | 78 | 27.3 | 24 | 33.3 | 0.141 | 365 | 31.7 |
|  | No | 95 | 72.0 | 434 | 65.8 | 208 | 72.7 | 48 | 66.7 |  | 785 | 68.3 |
| Periods | P1 | 19 | 14.4 | 150 | 22.7 | 82 | 28.7 | 25 | 34.7 | <0.0001 | 276 | 24.0 |
|  | P2 | 42 | 31.8 | 300 | 45.5 | 129 | 45.1 | 21 | 29.2 |  | 492 | 42.8 |
|  | P3 | 71 | 53.8 | 210 | 31.8 | 75 | 26.2 | 26 | 36.1 |  | 382 | 33.2 |

Supplementary Table 2: Axillary surgery for all patients and cN0 patients only. Abbreviations: cT, clinical tumor size stage; cN, clinical lymph node status; SLNB, sentinel lymph node biopsy; ALND, axillary lymph node dissection.

|  |  | **SLNB** | | **ALND** | | **SLNB+ALND** | | **Chi2** |
| --- | --- | --- | --- | --- | --- | --- | --- | --- |
|  |  | Nb | % | Nb | % | Nb | % | *p-value* |
| **all patients** | | 159 | 13.8 | 765 | 66.5 | 226 | 19.7 |  |
| Period | P1 | 3 | 1.9 | 208 | 27.2 | 65 | 28.8 | <0.0001 |
|  | P2 | 75 | 47.2 | 304 | 39.7 | 113 | 50.0 |  |
|  | P3 | 81 | 50.9 | 253 | 33.1 | 48 | 21.2 |  |
| T subtypes | Luminal A | 25 | 15.7 | 226 | 29.5 | 68 | 30.1 | <0.0001 |
|  | Luminal B-like HER2-negative | 12 | 7.5 | 107 | 14.0 | 38 | 16.8 |  |
|  | Luminal B-like HER2-positive | 24 | 15.1 | 127 | 16.6 | 42 | 18.6 |  |
|  | HER2-positive | 18 | 11.3 | 105 | 13.7 | 23 | 10.2 |  |
|  | Triple Negative | 80 | 50.3 | 198 | 25.9 | 54 | 23.9 |  |
|  | Luminal HER2-negative Grade? | 0 | 0 | 2 | 0.3 | 1 | 0.4 |  |
| cN stage | cN0 | 147 | 92.5 | 196 | 25.6 | 145 | 64.2 | <0.0001 |
|  | cN1 | 11 | 6.9 | 552 | 72.2 | 78 | 34.5 |  |
|  | cNx | 1 | 0.6 | 17 | 2.2 | 3 | 1.3 |  |
| cT stage | cT0-1 | 24 | 15.1 | 87 | 11.4 | 21 | 9.3 | <0.0001 |
|  | cT2 | 124 | 78.0 | 368 | 48.1 | 168 | 74.3 |  |
|  | cT3 | 11 | 6.9 | 239 | 31.2 | 36 | 15.9 |  |
|  | cT4 | 0 | 0 | 71 | 9.3 | 1 | 0.4 |  |
| Breast | Conservative | 127 | 79.9 | 254 | 33.2 | 110 | 48.7 | <0.0001 |
| surgery | Mastectomy | 32 | 20.1 | 511 | 66.8 | 116 | 51.3 |  |
| **only cN0 patients (n=488)** | | 147 | 30.1 | 196 | 40.2 | 145 | 29.7 |  |
| Period | P1 | 3 | 2.0 | 69 | 35.2 | 46 | 31.7 | <0.0001 |
|  | P2 | 69 | 46.9 | 54 | 27.6 | 65 | 44.8 |  |
|  | P3 | 75 | 51.0 | 73 | 37.2 | 34 | 23.4 |  |
| T subtypes | Luminal A | 25 | 17.0 | 70 | 35.7 | 43 | 29.7 | 0.001 |
|  | Luminal B-like HER2-negative | 12 | 8.2 | 22 | 11.2 | 24 | 16.6 |  |
|  | Luminal B-like HER2-positive | 22 | 15.0 | 28 | 14.3 | 28 | 19.3 |  |
|  | HER2-positive | 17 | 11.6 | 27 | 13.8 | 14 | 9.7 |  |
|  | Triple Negative | 71 | 48.3 | 49 | 25.0 | 36 | 24.8 |  |
| cT stage | cT0-1 | 22 | 15.0 | 29 | 14.8 | 16 | 11.0 | <0.0001 |
|  | cT2 | 116 | 78.9 | 95 | 48.5 | 106 | 73.1 |  |
|  | cT3 | 9 | 6.1 | 62 | 31.6 | 22 | 15.2 |  |
|  | cT4 | 0 | 0 | 10 | 5.1 | 1 | 0.7 |  |
| Breast | Conservative | 119 | 81.0 | 65 | 33.2 | 70 | 48.3 | <0.0001 |
| surgery | Mastectomy | 28 | 19.0 | 131 | 66.8 | 75 | 51.7 |  |

Supplementary Table 3: pCR rates according to tumor subtypes combined with cT and cN stages. Abbreviations: cT, clinical tumor size stage; cN, clinical lymph node status; pCR, pathologic complete response.

|  |  |  | **pCR** |  | **no pCR** | Chi 2 |  |
| --- | --- | --- | --- | --- | --- | --- | --- |
|  |  |  | Nb | % pCR | Nb | *p-value* | % pCR |
| Luminal A | cN0 | cT0-1 | 2 | 9.1 | 20 | 0.860 | 11.6 |
|  |  | cT2 | 11 | 13.1 | 73 |  |  |
|  |  | cT3 | 3 | 10.3 | 26 |  |  |
|  |  | cT4 | 0 | 0 | 3 |  |  |
|  | cN1 | cT0-1 | 2 | 10.5 | 17 | 0.622 | 5.8 |
|  |  | cT2 | 5 | 5.8 | 81 |  |  |
|  |  | cT3 | 3 | 5.8 | 49 |  |  |
|  |  | cT4 | 0 | 0 | 16 |  |  |
| Lum B Her2- | cN0 | cT0-1 | 1 | 9.1 | 10 | 0.332 | 22.4 |
|  |  | cT2 | 9 | 23.7 | 29 |  |  |
|  |  | cT3 | 3 | 42.9 | 4 |  |  |
|  |  | cT4 | 0 | 0 | 2 |  |  |
|  | cN1 | cT0-1 | 3 | 33.3 | 6 | 0.308 | 22.1 |
|  |  | cT2 | 13 | 26.0 | 37 |  |  |
|  |  | cT3 | 3 | 10.3 | 26 |  |  |
|  |  | cT4 | 2 | 28.6 | 5 |  |  |
| Lum B Her2+ | cN0 | cT0-1 | 3 | 33.3 | 6 | 0.228 | 48.7 |
|  |  | cT2 | 25 | 45.5 | 30 |  |  |
|  |  | cT3 | 9 | 69.2 | 4 |  |  |
|  |  | cT4 | 1 | 100 | 0 |  |  |
|  | cN1 | cT0-1 | 3 | 27.3 | 8 | 0.542 | 36.8 |
|  |  | cT2 | 23 | 38.3 | 37 |  |  |
|  |  | cT3 | 14 | 42.4 | 19 |  |  |
|  |  | cT4 | 2 | 20.0 | 8 |  |  |
| HER2-positive | cN0 | cT0-1 | 4 | 66.7 | 2 | 0.837 | 60.3 |
|  |  | cT2 | 24 | 63.2 | 14 |  |  |
|  |  | cT3 | 6 | 50.0 | 6 |  |  |
|  |  | cT4 | 1 | 50.0 | 1 |  |  |
|  | cN1 | cT0-1 | 3 | 50.0 | 3 | 0.382 | 55.7 |
|  |  | cT2 | 23 | 50.0 | 23 |  |  |
|  |  | cT3 | 13 | 56.5 | 10 |  |  |
|  |  | cT4 | 10 | 76.9 | 3 |  |  |
| Triple Negative | cN0 | cT0-1 | 17 | 36.8 | 12 | 0.365 | 46.8 |
|  |  | cT2 | 53 | 52.0 | 49 |  |  |
|  |  | cT3 | 12 | 37.5 | 20 |  |  |
|  |  | cT4 | 1 | 33.3 | 2 |  |  |
|  | cN1 | cT0-1 | 8 | 44.4 | 10 | 0.117 | 36.3 |
|  |  | cT2 | 35 | 38.9 | 55 |  |  |
|  |  | cT3 | 11 | 23.4 | 36 |  |  |
|  |  | cT4 | 7 | 53.8 | 6 |  |  |

Supplementary Table 4: Pathologic breast tumor response (ypT0-is) according to pN status, for all patients and for each tumor subtypes. Abbreviations: ypT, pathologic tumor status after neoadjuvant chemotherapy; ypN, pathologic nodal status after neoadjuvant chemotherapy; pN, pathologic lymph node status.

|  |  | **ypT0-is** |  | **ypT>=1** |  | **Chi2** |
| --- | --- | --- | --- | --- | --- | --- |
|  |  | Nb | % | Nb | % | *p-value* |
| Luminal A | | 49 | 15.4 | 270 | 84.6 |  |
| pN status | pN0sn | 3 | 6.1 | 21 | 7.8 | 0.014 |
|  | ypN0 | 23 | 46.9 | 71 | 26.3 |  |
|  | ypN1 | 23 | 46.9 | 178 | 65.9 |  |
| Luminal B-like HER2-negative | | 49 | 31.2 | 108 | 68.8 |  |
| pN status | pN0sn | 4 | 8.2 | 9 | 8.3 | 0.001 |
|  | ypN0 | 31 | 63.3 | 35 | 32.4 |  |
|  | ypN1 | 14 | 28.6 | 64 | 59.3 |  |
| Luminal B-like HER2-positive | | 94 | 48.7 | 99 | 51.3 |  |
| pN status | pN0sn | 11 | 11.7 | 14 | 14.1 | <0.0001 |
|  | ypN0 | 69 | 73.4 | 42 | 42.4 |  |
|  | ypN1 | 14 | 14.9 | 43 | 43.4 |  |
| HER2-positive ER-negative | | 94 | 64.4 | 52 | 35.6 |  |
| pN status | pN0sn | 13 | 13.8 | 5 | 9.6 | <0.0001 |
|  | ypN0 | 71 | 75.5 | 19 | 36.5 |  |
|  | ypN1 | 10 | 10.6 | 28 | 53.8 |  |
| Triple negative | | 164 | 49.4 | 168 | 50.6 |  |
| pN status | pN0sn | 44 | 26.8 | 37 | 22.0 | <0.0001 |
|  | ypN0 | 95 | 57.9 | 60 | 35.7 |  |
|  | ypN1 | 25 | 15.2 | 71 | 42.3 |  |
| Total | | 452 | 39.3 | 698 | 60.7 |  |
| pN status | pN0sn | 75 | 16.6 | 86 | 12.3 | <0.0001 |
|  | ypN0 | 290 | 64.2 | 227 | 32.5 |  |
|  | ypN1 | 87 | 19.2 | 385 | 55.2 |  |

Supplementary Table 5: OS, RFS and MFS rates with number of patients at risk and number of events. Abbreviations: OS, Overall survival; RFS, recurrence free survival; MFS, metastases free survival; SE, standard error; Nb, number.

|  |  | % | SE | Nb at risk | Nb events |
| --- | --- | --- | --- | --- | --- |
| OS | 2-years | 95.9 | 0.6 | 1032 | 45 |
|  | 5-years | 86.1 | 1.1 | 652 | 138 |
|  | 7-years | 81.0 | 1.4 | 343 | 168 |
|  | 10-years | 74.6 | 1.9 | 140 | 187 |
| RFS | 2-years | 89.1 | 0.9 | 958 | 122 |
|  | 5-years | 76.2 | 1.3 | 585 | 247 |
|  | 7-years | 73.2 | 1.5 | 339 | 266 |
|  | 10-years | 65.8 | 2.1 | 101 | 285 |
| MFS | 2-years | 90.4 | 0.9 | 969 | 107 |
|  | 5-years | 78.6 | 1.3 | 596 | 221 |
|  | 7-years | 75.7 | 1.4 | 348 | 239 |
|  | 10-years | 69.3 | 2.0 | 110 | 256 |

Supplementary Table 6. Survival results in univariate analysis. Abbreviations: OS, Overall survival; RFS, recurrence free survival; MFS, metastases free survival; cT, clinical tumor size stage; cN, clinical lymph node status; pCR, pathologic complete response; ypT, pathologic tumor status after neoadjuvant chemotherapy; ypN, pathologic nodal status after neoadjuvant chemotherapy; pN, pathologic lymph node status.

| Log Rank | OS | RFS | MFS |
| --- | --- | --- | --- |
| age | <0.0001 | 0.003 | 0.013 |
| cN status | <0.0001 | <0.0001 | <0.0001 |
| cT stage | 0.001 | 0.011 | 0.008 |
| pNsn/ypN | <0.0001 | <0.0001 | <0.0001 |
| ypT0-is vs ypT>=1 | <0.0001 | <0.0001 | <0.0001 |
| pCR vs no pCR | <0.0001 | <0.0001 | <0.0001 |
| Periods | 0.284 | 0.703 | 0.550 |
| Subtypes | <0.0001 | 0.016 | 0.024 |
| ER | <0.0001 | 0.071 | 0.340 |

Supplementary Figure 1: Flow chart.
